# Collectanea Medica: Consisting of Anecdotes, Facts, Extracts, Illustrations, &c.

**Published:** 1824-12

**Authors:** 


### COLLECTANEA MEDICA:

CONSISTING OF

### ANECDOTES, FACTS, EXTRACTS, ILLUSTRATIONS, &c.

***Relating to the History or the Art of Medicine, and the Collateral Sciences.***

---

Floriferis, ut apes, in saltibus omnia libant,
Omnia nos, itidem, depascimur aurea dicta.

---

ART. I.—*Notice of a Mode by which a Conjecture may be formed as to the Sex of the Chick in Ovo.* By Mr. DAVID RITCHIE. (From the Edinburgh Philosophical Journal.)

IN physiology, it is interesting to ascertain at what period the embryo assumes a sex, and by what means its sex may be distinguished. In regard to viviparous animals, such inquiries are attended with much difficulty, as accurate dissection and minute microscopic observation are necessary. Such investigations, too, cannot be carried on without cruelty. Hence it is that physiologists have so often and so carefully observed and described the embryo in oviparous animals, from its first appearance in the egg till its complete development. For here the embryo may be subjected to examination without injury to the parent, and there is such similarity in the mode of growth of animals in the one case and in the other, that any fact ascertained as to the chick in the egg has usually led to a similar discovery in regard to the fœtus in utero. The appearances presented by the egg have been described by the most celebrated anatomists of our own and of other countries. I shall advert to the opinions which have been entertained by them as to the egg, only in so far as they relate to my present subject.

Eggs of the same bird differ from each other in shape. It has been asserted that the longer eggs contain males, the shorter ones females. To determine this, experiments were lately made by Dr. Autenreith, jun. of Tubingen. From his experiments it appears, "that the opinion so generally entertained by his countrymen, that cocks are contained in the longer eggs, and hens in the shorter and rounder ones, is without foundation; for the sex is indicated neither by the shape nor by the size, nor in any way by the external form of eggs."

A French naturalist, Bory de St. Vincent, speaks of a physician who had examined many thousand eggs, without being able to ascertain, from their external appearance, whether they contained cocks or hens.

Eggs of the same bird differ from each other in specific gravity. It has been conjectured, that the heavier eggs may contain males, the lighter ones females. But it is now generally known that eggs lose weight by being kept; and it has been shewn that this diminution arises from "the substitution of air for a portion of the water of the egg which escapes."

Eggs were found by Dr. Prout to differ from each other in the relative proportions of their saline principle. "I have sometimes thought," says he, " these differences, as well as some other singular ones observed with respect to the earthy matters, might be connected with the sex of the future bird :" but he adds, " no proof of this could be obtained."

I have heard of a mode of discovering the sex of the chick in ovo, different from any which has been proposed by naturalists. The folliculus aëris, or air-cell, which is to furnish oxygen to the futue chick, is situated at the larger end of the egg. It has not in all eggs the same position at the larger end; and, in various districts of Scotland, it is believed that eggs having the air-cell situated *exactly* at the top of the larger end produce males, while those having the air-cell only *near* the top of the larger end produce females.

To ascertain this, I instituted a series of experiments. These experiments, as will be seen, go very far to prove that the opinion which has been stated is correct, and so to determine what naturalists of Germany, France, and England, have endeavoured in vain to discover.

Eggs were selected, all having the air-cell situated exactly on the apex of the larger end, and were hatched. In most instances, the birds were all males.

Eggs were selected, all having the air-cell situated, not on, but near to the apex of the larger end. In most instances, the birds were all females.

These experiments have been frequently repeated, and were conducted for me by persons residing in different parts of the country.

A few of the experiments were not quite so satisfactory as the others. Among a dozen of birds of one brood, two or three turned out to be of a different sex from the rest. I may remark, however, that it is sometimes rather difficult to determine whether the air-cell be exactly on the apex or not; and the anomaly may have originated in error arising from this cause.

Every person has observed the air-cell in the boiled egg upon removing the shell: in the recent egg, it may be perceived by holding the egg between the eye and a lighted candle. In general, even an unpractised observer may determine the position of the cell, whether it be at the axis or some little way from it, and so conjecture the sex of the future bird. Occasionally, however, as already remarked, it is rather difficult to decide whether the air-cell is or is not on the axis. Such eggs should not be used by those who may be induced to put the criterion to the test of experiment.

The fact ascertained by these experiments seems to deserve the attention of the economist and of the physiologist.

At present I would only remark, that the result militates against an opinion, which has been ably defended, in regard to the ova of quadrupeds, and which has been advanced, too, as to the eggs of birds,—that, previous to impregnation, they have no distinction of sex, but are so formed as to be equally fitted to become male or female; and that it is the process of impregnation which marks the distinction.

The air-cell exists in unfecundated eggs. In some of these it is situated at the top of the larger end; in others, near the top. From this it would appear that the male parent does not influence the sex of the embryo, but that the ovum has assumed its sex previous to impregnation.

Art. II.—*On the Detection of minute Quantities of Arsenic in mixed Fluids.* By Robert Christison, M.D. F.R.S. Professor of Medical Jurisprudence in the University of Edinburgh.—(From the Edinburgh Philosophical Journal.)

The object of this paper is to estimate the value of the liquid tests for arsenic, when dissolved in mixed vegetable and animal fluids, and of the various processes which have been proposed for correcting the changes thus produced in the action of the tests; and, finally, to determine what mode of analysis is at the same time the simplest, and may be applied under all circumstances. The author has extended the researches of Orfila, and those on the modifications caused in the action of the tests, by the co-existence of animal and vegetable fluids; and shows, by the instances of broth, tea, coffee, porter, port wine, and milk, that, when the fluid is complicated or deeply coloured, and the arsenical solution of such moderate strength as may be usually looked for in medico-legal investigations, the four best liquid tests,—namely, lime-water, the ammoniacal sulphate of copper, the ammoniacal nitrate of silver, and sulphuretted hydrogen, are almost or absolutely useless. He next proves that no advantage is to be derived from the plans which have been proposed for restoring the true action of the tests, by destroying the colour of the fluids. These are two in number: the one advanced by Orfila, in 1821; the other by Mr. Phillips, in January last. The former chemist proposes to destroy the colours by chlorine; the latter, by digestion with animal charcoal. But Dr. Christison finds that, after the action of chlorine, the colour is seldom altogether or sufficiently destroyed; that the process does not take from the fluid the power it often possesses, of retaining the arsenical precipitate in solution; and that, in some decolorised fluids, containing no arsenic, some of the tests cause precipitates very similar to those produced in pure arsenical solutions. He likewise finds, that the process by digestion in charcoal is sufficient, because, if the solution is not very strong, the charcoal removes all, or nearly all, the arsenic, as well as the colouring matter; and, if the solution be strong, it does not always lose its property of retaining the arsenical precipitates dissolved. He then proceeds to examine the processes which have been recommended by Rose and Rapp for discovering arsenic, when intimately mingled with the animal textures, and which might also be applied to the residue by evaporation of mixed fluids, in which the common tests do not act characteristically. These processes it is unnecessary to mention. They are founded on the possibility of arsenic so combining with animal matters, as to resist the solvent power of boiling water. But Dr. Christison has found that, after careful digestion in water, no arsenic can be detected by either process; and, farther, that the process of Rapp is otherwise insufficient, when the quantity of arsenic is small, in which circumstance alone the ordinary means of analysis are inadequate.

In the last place, he describes the method which he has found easiest of execution, and most generally applicable, for detecting arsenic dissolved in mixed fluids, or mingled with such solids as are incapable of forming with it an insoluble compound. Minute directions are given, for the sake of the inexperienced; but we shall notice only the essential parts, and the result of his experience with respect to the delicacy of the method. Having observed that the sulphuretted hydrogen, though it seldom acts characteristically on diluted solutions of arsenic in mixed fluids, nevertheless always throws it down of some colour or other, even when the proportion of poison does not exceed an 8000th part, he proposes to employ this test, with the view of procuring the arsenic in a convenient form for being subjected to the decisive process of reduction.

"The suspected matter, if solid, is to be divided into minute fragments, and boiled briskly in two or three successive portions of pure water. The fluid, whether originally such, or procured by digestion from the solid matter, is then to be subjected, in a deep, narrow glass, for half an hour, to a brisk stream of sulphuretted hydrogen gas. In many cases, however, it will be necessary to premise the two following preparatory steps, before transmitting the gas; and, as we can seldom know before-hand whether these steps are requisite or not, it may be right to resort to them in every case. The first precaution is to add a little acetic acid to the fluid. By so doing, the influence of any free alkali that may exist in it is counteracted; and several organic principles, which might impede the subsequent separation of the precipitate, are coagulated. The second precaution is to boil the fluid for a few minutes; by which means some matters are separated, that the acetic acid could not throw down entirely; and any carbonic acid existing in it is driven off. The presence of carbonic acid in considerable quantity, by impeding the solution of the sulphuretted hydrogen, prevents its action on the arsenic, if the proportion of arsenic be small. The fluid is then to be filtered."

"When the stream has been continued a sufficient length of time, there is either a precipitate formed, or the fluid acquires a yellowish milkiness, which passes to a distinct precipitate as soon as the excess of sulphuretted hydrogen is driven off by heat. It is always right to boil, before attempting to separate the matter thrown down; as the precipitate then becomes much more distinct, and falls to the bottom more readily. When the filtration is finished, and the filter has been gently compressed between several folds of bibulous paper, the precipitate is to be scraped off with a knife, and dried on a bit of smooth paper, at a temperature somewhat above 212°."

The most advisable mode of subjecting this to the test of reduction, is the following:—The best flux is the black flux, and the best instrument a glass tube, closed at one end, open at the other, about three inches long, and varying from a fourth to an eighth of an inch diameter, according to the bulk of the material, which should not fill above two-fourths of an inch of the tube. The best mode of applying heat is by the alcohol lamp, as recommended by Mr. Phillips.

"The true arsenical crust is known by the following physical character:—Its outer surface next the tube exactly resembles highly polished steel. Its inner surface (which is best seen by scratching the tube with a file at the lower margin of the crust, and snapping it across,) is precisely like the fracture of fine steel, if the quantity is considerable: if it is minute, it has a dull bluish-grey appearance, but, before a microscope of four or five powers, appears brilliant and crystalline, like the fracture of steel. Occasionally, when very minute in quantity, it appears botryoidal, and not brilliant, even before the microscope: in that case, the part of the tube to which it is attached should be coarsely powdered, and heated anew in a tube of less diameter. It is scarcely possible for any one to mistake these characters, particularly if he has ever seen an arsenical crust before; but, to prevent all possibility of error, the analysis may be concluded with the following experiment:—The part of the tube to which the crust is attached, being broken into fragments, is to be left for some hours in a watch-glass, containing a dilute solution of the ammoniacal sulphate of copper, and covered to prevent evaporation. In four or five hours, the metallic crust will become grass-green; or, if very minute, it will be discoloured, and a brilliant grass-green crust formed on the surface of the liquor. The simple evaporation of the fluid will cause the formation of a crust on its surface, though no arsenic be immersed in it. But in that case its colour is pale blue."*

The author concludes by stating, that the evidence thus procured is quite unimpeachable; that the process is the most convenient yet proposed; that it is probably applicable to all cases without exception, as he has found it to answer with the most complicated fluids he could select,—namely, broth, tea with cream and sugar, coffee similarly made, porter, port wine, and milk; and that it is sufficiently delicate for all medico-legal purposes, as it will detect satisfactorily a quarter of a grain of arsenic dissolved in 8000 parts of any of the foregoing fluids.

---

ART. III.—*On Fumigation.* By M. FARADAY, F.R.S. Corresponding Member of the Academy of Sciences of Paris, Chemical Assistant of the Royal Institution, &c.—(Journal of Science, &c.)

I WAS called on, some months since, to direct and superintend the fumigation of the General Penitentiary at Millbank; in doing which, some precautions and arrangements suggested themselves, which I have thought might be usefully made known for the information of those who may have occasion to apply disinfecting agents to the purification of buildings, either large or small.

On examining a building to be fumigated, it is necessary to estimate the surface exposed to the infectious vapours, as well as the capacity of the structure. When the air of a place is impregnated with infectious matter, the surface of the walls, &c. will absorb more or less of it in proportion as it is more or less extensive, as it approaches nearer to or is farther from the source of infection, and also in some degree according to its nature.

The general arrangement of the Penitentiary was favourable to its complete and perfect fumigation; for, though of great magnitude, yet its division into smaller parts, as galleries, towers, staircases, &c., most

* If portions of the crust are simply exposed to the air, they soon acquire a greyish-black colour on the surface; a character also indicative of metallic arsenic. —EDITOR *of the Phil. Journal.*)

of which were glazed, and all of which could be closed by doors so as to separate them from each other, rendered the successive application of the means employed easy and convenient.

After deciding upon fumigation by chlorine, the next object was to ascertain the most favourable mode of applying it; and I was desirous, for many reasons, of obtaining a gradual and successive development of the disinfecting agent, rather than a sudden and short one. The latter mode, though it would have filled the building at once, and probably very effectually, yet would seriously have incommoded the operators, and would also soon have disappeared, in consequence of absorption by the limed walls, and from dissipation through apertures that would inevitably remain unclosed in different parts of the building: whilst the former mode, by continually supplying the disinfecting agent to the atmosphere of the place for a length of time, would enable it better to act on the bedding, clothing, and other articles left in the cells, and allow it also more perfectly to penetrate to every part of the building itself.

The materials used were those generally employed,—namely, common salt, oxide of mangenese in powder, and oil of vitriol. Upon making experiments with these substances, as furnished by the dealer for the fumigation, I found that a mixture of one part, by weight, of common salt and one part of the oxide of manganese, when acted upon by two parts of oil of vitriol, previously mixed with one part, by weight, of water, and left till cold, produced the best results. Such a mixture, made at temperatures of 60° Fahr., liberated no muriatic acid; but in a few minutes began to evolve chlorine, and continued to do so for four days. When examined on the fifth day, and urged by heat, so as to cause the liberation of all the chlorine that could be afforded by it, only a small proportion was obtained. Such a mixture may, therefore, be considered as having liberated its chlorine gradually but perfectly, without the application of any extraneous heat; and is, therefore, very proper for extensive fumigation.

The vessels in which the mixture is to be made should be flat, and such as, being economical, are least acted on by the chlorine or acid. Common red pans were used in the Penitentiary; for, many being required at once, better earthenware would have been too expensive. They held each about four quarts.

Preparatory to the fumigation, a quantity of the salt was turned out, the lumps broken down by a mallet until the whole was in powder, and then an equal weight of the oxide of manganese added, and the whole well mixed. The acid and water were mixed in a wooden tub, the water being put in first, then about half the acid added, stirring at the same time. When the heat produced had been dissipated, which happened in a few hours, the rest of the acid was added, stirring as before, and the whole left till cold. The men used measures in mixing the acid and water, and were told to take rather less of water than of acid; nine measures to ten being nearly the quantities required. Any slight departure from these proportions would be of no consequence. The pans were then charged, each with about three pounds and an eighth of the mixed salt and manganese, and distributed at proper intervals along the galleries, &c.; care having been taken previously to close the doors and windows, and to stop with mats or rugs all apertures to which access could be had, especially key-holes, through which there was any draught. The diluted acid, being cold, was carried in cans or jugs, and measured out in the proportion of four pounds and a half to each pan, the mixture being well stirred with a stick, and left to itself. This was done without any inconvenience to the operator, except when the acid was applied too warm: there was abundant time to go from pan to pan, and to close the various galleries in succession. On entering a gallery a few minutes after it had been thus treated, the general diffusion of the chlorine in the atmosphere was sufficiently evident. In half an hour, it was often almost impossible to enter; and frequently, on looking along the gallery (150 feet in length), the yellow tint of the atmosphere could easily be perceived. Up to the fifth day, the odour of the chlorine could generally be observed in the building. After the sixth, the pans were removed, though sometimes with difficulty, to be emptied and used elsewhere; and the place fumigated had its windows and doors thrown open.

It was estimated that the charge of each pan would yield about one pound of chlorine, or five and a half cubical feet. The whole quantity of materials used was 700 pounds of common salt, 700 pounds of oxide of manganese, and 1400 pounds of oil of vitriol. The space requiring fumigation amounted to nearly 2,000,000 cubical feet; and the surface of the walls, floors, ceilings, &c. (exclusive of furniture, bedding, &c.) was about 1,200,000 square feet. This surface was principally stone and brick, most of which had been lime-washed. The space was divided into seventy-two galleries, of 150 feet each in length; and towers, passages, chapel, &c. equivalent to about thirteen galleries more. The number of cells, rooms, &c. was nearly 1200.

It was desirable, for many reasons, that the Penitentiary should be fumigated in the most unexceptionable manner; and the means employed were, therefore, applied to an extent probably far beyond that requisite to the destruction of any miasmata that might be within it. The proportion of chlorine evolved to the size and surface of the building may be considered, therefore, as sufficient for a case of the most excessive kind; and, though the limits are guessed at, rather than judged of by any well-founded rule, yet I should consider from one-half to one-fourth of the chlorine as quite sufficient for any of the usual cases where fumigation is required.

